# Urine Sediment Findings and the Immune Response to Pathologies in Fungal Urinary Tract Infections Caused by *Candida* spp.

**DOI:** 10.3390/jof6040245

**Published:** 2020-10-23

**Authors:** José Antonio Tesser Poloni, Liane Nanci Rotta

**Affiliations:** 1 Health School, Universidade do Vale do Rio dos Sinos, São Leopoldo 93022-750, Brazil; 2 Controllab, Rio de Janeiro 20911-442, Brazil; 3 Post-Graduation Program in Health Sciences Diagnostic Method Department, Universidade Federal de Ciências da Saúde de Porto Alegre, Porto Alegre 90050-170, Brazil; lnrotta@ufcspa.edu.br

**Keywords:** urine sediment, urinary tract infection, *Candida* spp., fungal infection, immune response

## Abstract

Fungi are pathogenic agents that can also cause disseminated infections involving the kidneys. Besides *Candida*, other agents like *Cryptococcus* spp. can cause urinary tract infection (UTI), as well as other non-yeast fungi, especially among immunocompromised patients. The detection and identification of fungi in urine samples (by microscopy and culture) plays an essential role in the diagnosis of fungal UTI. However, variable cutoff definitions and unreliable culture techniques may skew analysis of the incidence and outcome of candiduria. The sediment analysis plays a key role in the identification of fungal UTI because both yeasts and pseudohyphae are easily identified and can be used as a clinical sign of fungal UTI but should not be overinterpreted. Indeed, urine markers of the immune response (leukocytes), urine barriers of tissue protection (epithelial cells), and urine markers of kidney disease (urinary casts) can be found in urine samples. This work explores the manifestations associated with the fungal UTI from the urinalysis perspective, namely the urinary findings and clinical picture of patients with fungal UTI caused by *Candida* spp., aspects associated with the immune response, and the future perspectives of urinalysis in the diagnosis of this clinical condition.

## 1. Introduction

Fungal urinary tract infection usually means urinary tract infection (UTI) caused by *Candida* species with special attention to *Candida albicans* as the major fungal infectious agent [1]. Fungi are pathogenic agents that can also cause disseminated infections involving the kidneys. Besides *Candida*, other agents, such as *Cryptococcus* spp., can cause UTIs. Non-yeast fungi, like some members of the *Aspergillus*, *Mucorales*, *Blastomyces*, and *Histoplasma* families, can also cause infections, especially among immunocompromised patients [2]. The detection and identification of fungi in urine samples (by microscopy and culture) plays an essential role in the diagnosis of fungal UTI. Leukocyturia is a cardinal sign of UTI caused both by bacteria or fungi [3,4] and also is a key sign of the immune response occurring in the urinary tract when the organism faces an UTI. This work explains the urinary findings and clinical picture of patients with fungal UTI caused by *Candida* spp. and some aspects related to the immune response in fungal UTI, in addition to urinalysis perspectives in this clinical condition.

## 2. Fungal Urinary Tract Infection

Urinary infections by *Candida* (usually asymptomatic) are caused by either the ascending or the hematogenous routes. By the ascending route, fungi are suggestively derived from the perineum, spreading into the bladder and then leading to colonization (it can occur into the collecting system of the kidneys) [2,5,6]. The ascending infection of the upper urinary tract is rare, and the risk increases with obstruction of it, diabetes mellitus, or urinary reflux [5,6]. The yeasts adhere to endothelial or urothelial cells, colonize the local area, evade the immune response, and, lastly, invade tissue or disseminate to distant sites within the body. The presence of an indwelling catheter allows biofilm formation and the persistence of the organism [2,7]. On the other hand, the hematogenous route is the way that the most kidney infection occurs in physiological state. *Candida* penetrates in the proximal tubules after passing by glomeruli and is eliminated in urine within weeks. Autopsy studies have noted renal cortical micro abscesses in most patients who have died of invasive candidiasis [2,8].

It is possible to clinically group patients whose urine presents *Candida* species: Patients with asymptomatic candiduria (previously healthy, outpatients), patients with asymptomatic candiduria (predisposed inpatients), patients with symptomatic candiduria (UTI), and clinically unstable patients with candiduria [9,10]. Once the presence of *Candida* in the urine is confirmed, careful clinical evaluation should be performed to detect symptoms or suggestive signs of other conditions, such as diabetes mellitus, genitourinary structural abnormalities, decreased renal function, and metabolic syndromes [9,10,11]. For patients with persistent candiduria, treating certain clinical conditions or removing risk factors is usually sufficient to eliminate the presence of *Candida* in the urine and no antifungal treatment is required [9].

## 3. Definition and Diagnosis of Candiduria and UTI Caused by *Candida* spp.

The definition of candiduria is enigmatic. Although most studies rely on culture, both urine microscopic visualization and culture of urine could be employed. Of concern is that neither the diagnostic criterion of colony forming unity (CFU) cutoff nor the collection technique (suprapubic aspiration versus bag collection) for neonatal urinary candidiasis is standardized. Even in adults, CFU criteria to diagnose candiduria range from 10^3^ to 10^5^ CFU/mL. In some studies, candiduria is even differentially defined for women and men [7,12]. In addition, in most retrospective studies, standard urine cultures were screened for candiduria, which means that urine was cultured on MacConkey and blood agar only [13]. Some laboratories have realized the urine cultures on Uriselect agar, which is a chromogenic agar that allows the preliminary perform identification of predominantly bacterial uropathogens [7,13]. Although these culture methods are certainly sufficient to identify bacteria, they may be significantly less sensitive to recover *C. albicans* and non-*C. albicans* species. Consistent with this concern, prospective studies in which urine was cultured on Sabouraud dextrose (SD) agar, a standard fungal medium, have reported higher numbers of non-*C. albicans* species [7,14,15]. It is noteworthy that the fungal burden could be relevant, because a statistically significant correlation between heavy candiduria (>10^4^ CFU/mL urine) and a high Pittet *Candida* colonization index (>0.5) has been established [7,16]. In summary, variable cutoff definitions and unreliable culture techniques may skew analysis of the incidence and outcome of candiduria. These discrepancies have not been adequately addressed in most studies [7].

### 3.1. Urinalysis

Urine examination plays a key role in the identification of fungal UTI. Two very basic tests can be cited here: Urine sediment analysis and urine culture. This review focused on the urine sediment analysis a test that strongly contributes to the detection of fungal UTI, because both yeasts and pseudohyphae are easily identified on direct examination of the sample between slide and cover slide [3,4]. Also, urine markers of an immune response (leukocytes), urine barriers of tissue protection (epithelial cells: squamous, transitional, and renal tubular), and urine markers of kidney disease (urinary casts) can be found in urine samples.

#### 3.1.1. Urinary Particles

**Figure 1:**
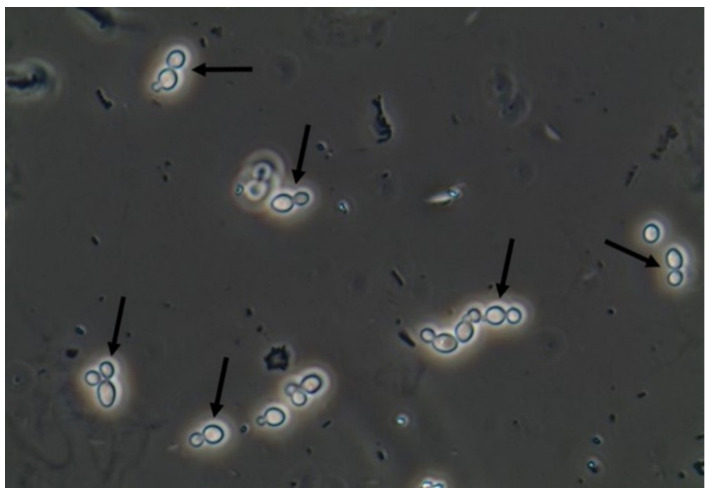
*Candida albicans* yeasts in the fresh and unstained urine sediment. Phase contrast microscopy. Original magnification: 400×. Courtesy: Controllab.

**Figure 2:**
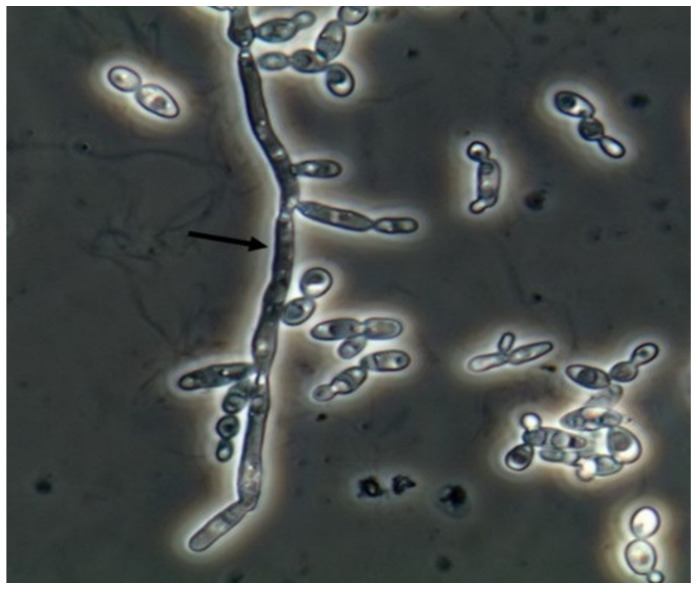
*Candida albicans* pseudohyphae in the fresh and unstained urine sediment. Phase contrast microscopy. Original magnification: 400×. Courtesy: Controllab.

**Figure 3:**
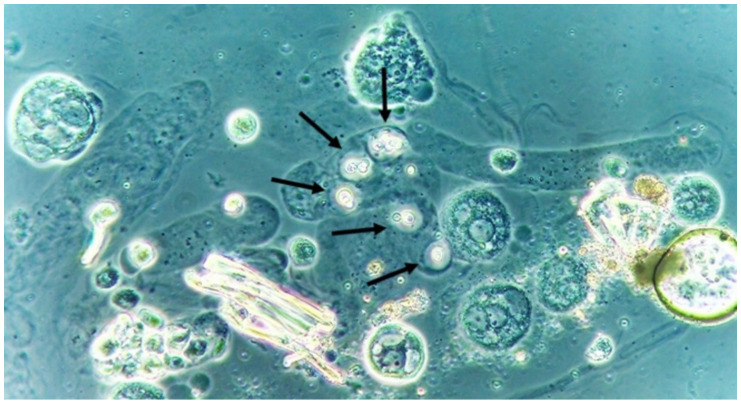
*Cryptococcus neoformans* encapsuled yeasts within a urinary cast. Fresh and unstained urine sediment. Phase contrast microscopy. Original magnification: 400×.

Yeasts (Figure 1) are observed, without the necessity of any special stain, usually at 400× magnification, as pale-green cells with smooth and well-defined walls. The nucleus can sometimes be seen, and the cytoplasm is homogenous without apparent organelles. Usually, the shape of the yeast cells is ovoid, spherical, or elongated. If urine stands for long periods at the bench, abundant pseudohyphae (Figure 2) can be seen [17]. In an infection condition, yeasts in urine may reflect invasive fungal infection, which may cause urethritis, cystitis, or renal infection. In renal infections caused by yeasts, casts containing these structures can be observed. Certain clinical conditions are more frequently linked to fungal UTI, such as diabetes mellitus, structural abnormalities of the urinary tract, indwelling catheters, prolonged antibiotic treatment, or immunosuppression [4]. The casts containing fungi are structures that deserve attention. Urinary casts are unique structures, exclusively produced within the tubular lumen under some circumstances (e.g., low intratubular pH, high osmolality, and high sodium concentration). Urinary casts can have several structures attached to their proteinaceous matrix: Erythrocytes, leukocytes, renal tubular epithelial cells, lipids, bacteria, and fungi. In fact, any structure passing through the tubular lumen during the formation of the cast can be attached to the Tamm-Horsfall fibrils (that forms the cast), and when the cell or microorganisms are seen within an urinary cast, they are a clue to the presence/passage of this cell or microorganism within/through the kidneys [3,4,18]. Not only *Candida* spp. yeasts were observed within casts [18], but *Cryptococcus* spp. (Figure 3) were already reported [19], helping in the identification of fungal UTI caused by this pathogenic agent particularly important to immunocompromised patients. Detection of renal *Candida* casts may be a useful diagnostic marker in distinguishing upper versus lower urinary tract candidiasis.

**Figure 4:**
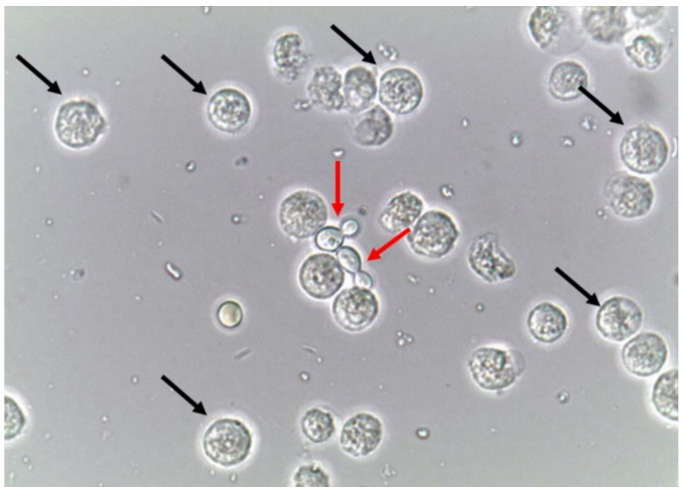
Leukocytes (some of them pointed with black arrows) and yeast cells (red arrows). Fresh and unstained urine sediment. Bright field microscopy. Original magnification: 400×. Courtesy: Controllab.

**Figure 5:**
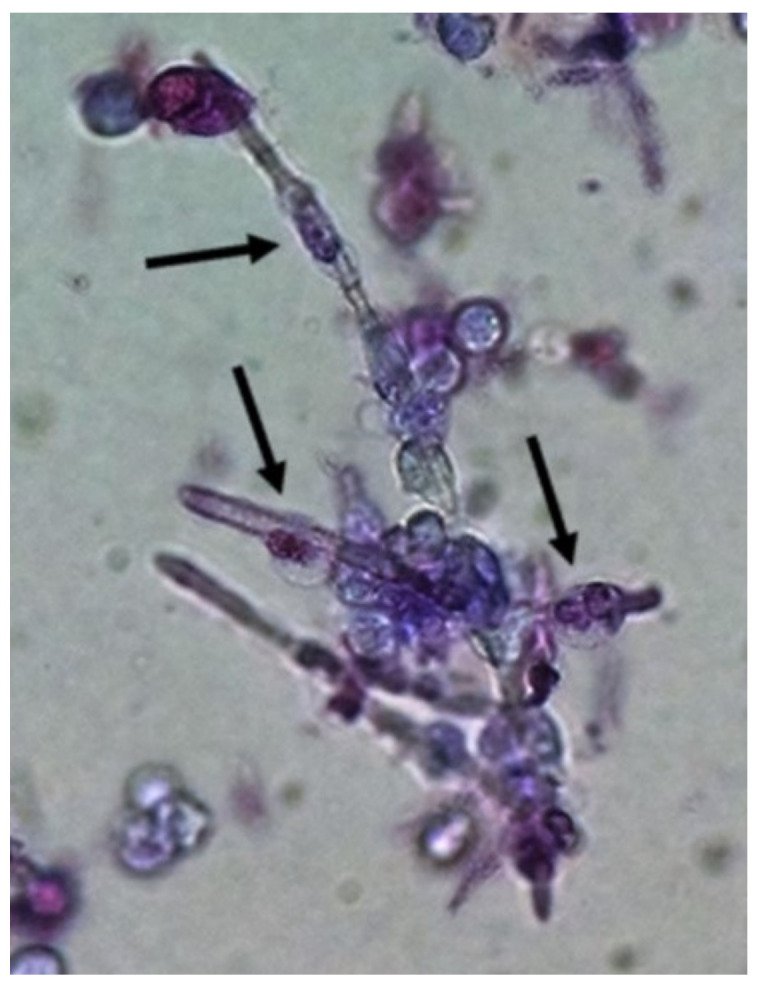
Neutrophils (some of them pointed) firmly attached to *Candida albicans* pseudohyphae presumably trying to perform phagocytosis of the fungal structure. Urine sediment stained with Sternheimer-Malbin stain. Bright field microscopy. Original magnification: 400×.

Due to the nature of the infectious process, leukocytes (granulocytes, particularly neutrophils) (Figure 4) are easily observed in the urine sediment where fungal structures are found. They can be observed in small numbers or in large amounts. Sometimes, they can be observed trying to perform phagocytosis of pseudohyphae (Figure 5). They can reflect an infectious/inflammatory process [3,4].

**Figure 6:**
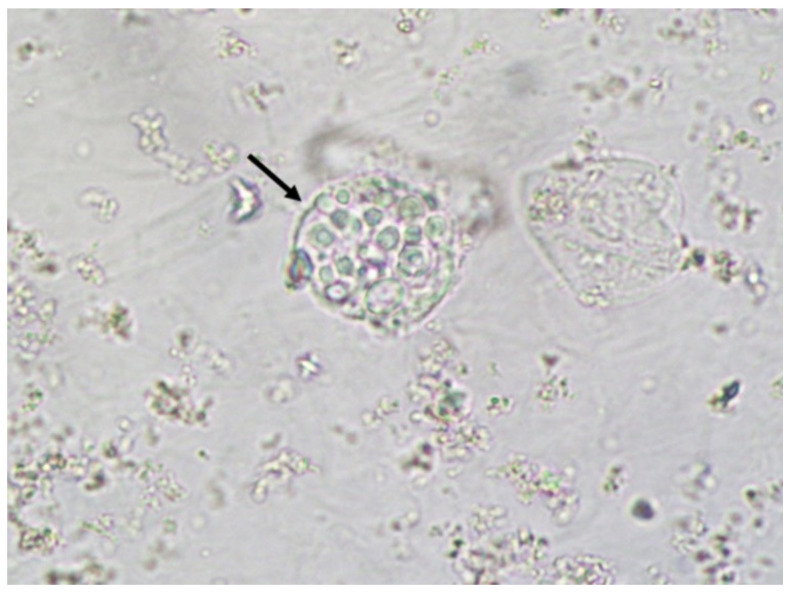
Macrophage with *Cryptococcus neoformans* yeasts engulfed. Fresh and unstained urine sediment. Bright field microscopy. Original magnification: 400×.

Macrophages are efficient phagocytes and were already reported engulfing yeast in urine sediment samples (Figure 6) [20]. Macrophages can appear on urine samples due to inflammation/infection of any tissue from the urinary tract, from the kidneys to the urethra. Thus, the finding of macrophages in urine samples and even macrophages with fungal particles engulfed only reflects the function of this kind of cell in the genitourinary tract [20].

Epithelial cells are a very important component of the body’s defensive system, since they are the first barrier to block the pathogenic agents to enter and cause infection. 

**Figure 7:**
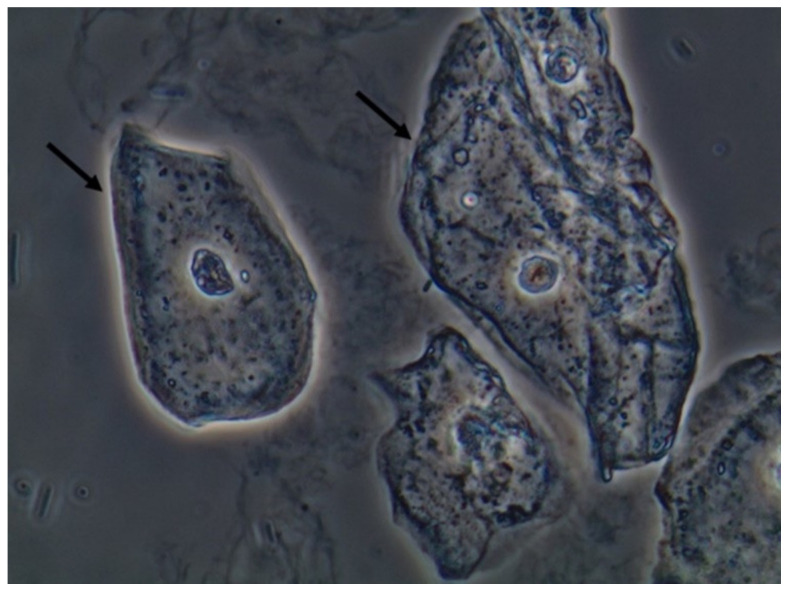
Squamous epithelial cells in the fresh and unstained urine sediment. Phase contrast microscopy. Original magnification: 400×. Courtesy: Controllab.

Squamous epithelial cells (Figure 7) compose the first layer of defense where the mucosa is present, such as, for example, the urethra. This type of cell is permanently renewed and, if the patient does not perform adequate hygiene before the urine sample collection, a large number of this cell type will be observed in urine sediment. This is important to mention because they are also present in the vagina and any part covered with mucosa. Their finding in large numbers in the sample is a clear sign of an inappropriate sample collection procedure. It is not considered a pathological finding, since bodies continuing to replace it to keep the epithelial barrier in full action [3,4].

**Figure 8:**
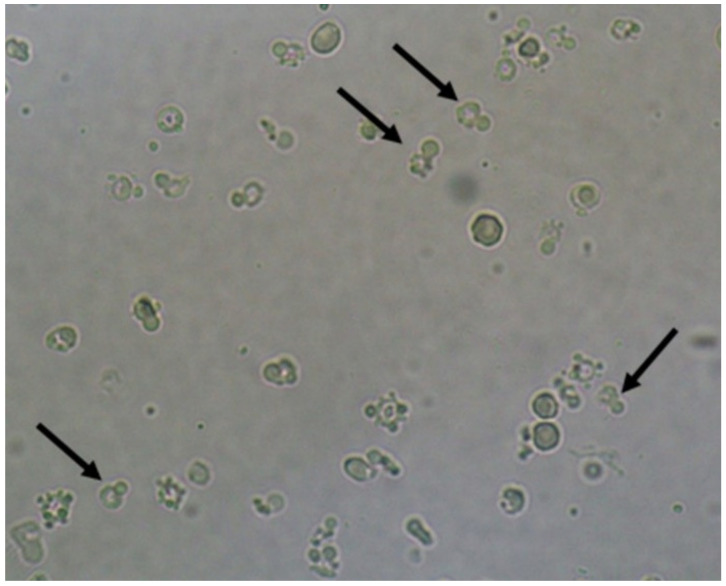
Acanthocytes (some of them pointed). Fresh and unstained urine sediment. Bright field microscopy. Original magnification: 400×.

**Figure 9:**
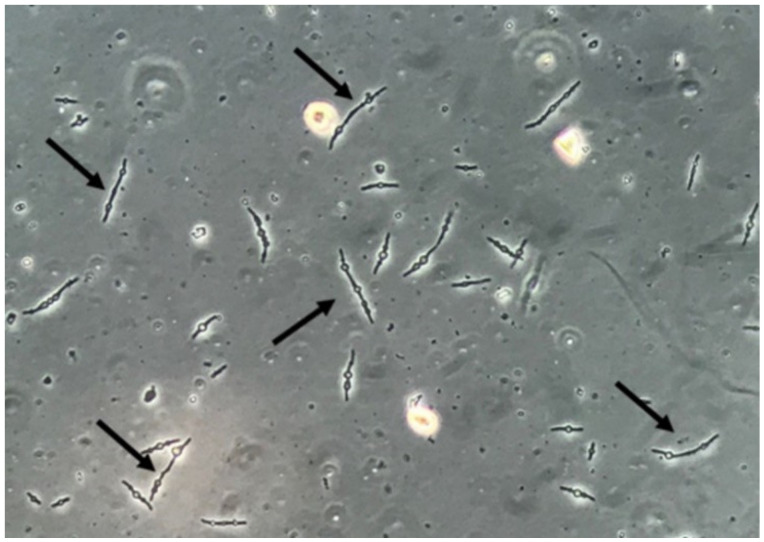
Multiresistant *Escherichia coli* bacteria forming filamentous forms and spheroplasts. Fresh and unstained urine sediment. Phase contrast microscopy. Original magnification: 400×.

It is important to mention that yeast cells observed in urine sediment can present a morphological resemblance with erythrocytes, lipid droplets, calcium oxalate monohydrate crystals, and, especially, acanthocytes (a particular type of dysmorphic erythrocytes that presents blebs protruding from the cell membrane due to the passage through the glomerulus and tubular system) (Figure 8). Professionals performing the microscopic evaluation of urine sediments need to receive proper training and use good-quality microscopes to avoid misidentification of these particles [21]. Indeed, bacteria can be found deformed in urine sediment, presenting elongated, thin, and filamentous forms, sometimes with a swollen and ball-like part of each bacteria, called spheroplasts (Figure 9). These spheroplasts can be seen after the use β-lactamic antibiotics [22,23]. Both filamentous forms and spheroplasts usually are seen with sizes much larger than bacteria usually presents, potentially leading to misidentification of these bacterial-deformed forms as fungal structures.

#### 3.1.2. Urine Sediment Profile on Fungal UTI

In the fungal UTI, the urine sediment examination should present, basically, yeasts and/or pseudohyphae besides leukocytes. Immune cells (macrophages or neutrophils), performing phagocytosis of urinary fungal particles and casts containing fungi, are possible, but are an extremely rare microscopic finding. Erythrocytes, renal tubular epithelial cells and urinary casts can be observed if the fungal UTI (or other related clinical conditions that can happen concomitantly with the fungal UTI) leads the patient to a more aggressive injury in the renal tissue. These findings can be linked to the loss of kidney function [3,4].

The urinary finding of yeasts and pseudohyphae can be a clue to the identification of fungal UTI. Surely, this is the main information that suggests whether the urine sediment can contribute to this kind of clinical condition. Indeed, the test is easy and fast to perform, and the fungal structures are easy to observe under the microscope. Fungal structures in the urine sediment can be used as a clinical sign of fungal UTI but should not be overinterpreted. The observation of yeasts and pseudohyphae can also be due to sample contamination, mainly when the sample is improperly collected.

The quality of the urine sample collection depends on factors such as the proper collection instruction furnished by the laboratory staff and the comprehension by the patient of the necessity to collect the sample according to the instructions of the laboratory (observing the clean catch technique). The evaluation of the urine sample under the microscope should take place within 2 h of sample collection.

The main difficulty of the interpretation of the fungal structures observation in the urine sediment is the fact that this finding can be both a structure with diagnostic value and a sample contaminant. Also, there is no information in the literature that has properly defined the differentiation between these antagonic situations.

## 4. Clinical Presentation of Candiduria

Fungal UTIs are mostly asymptomatic [7,24]. Leukocyturia, which is also not part of the definition criteria of asymptomatic bacteriuria, is mostly not present in candiduric patients. *C. albicans* UTIs are commonly associated with the use of catheters [7,24,25]. Candiduria occurs late in the hospital stay. In a large prospective study done in French intensive care unities (ICUs), the mean interval between ICU admission and candiduria was 17.2 ± 1.1 days, and similar numbers were reported in studies from Spain [26,27]. In renal transplanted patients, the first episode occurred a median of 54 days after transplantation (range: 0 to 2922 days) [28]. In these patients, candiduria is also mostly asymptomatic. Candiduria can be the result of uncomplicated cystitis and/or pyelonephritis, analogous to bacteriuria. Studies have reported that a low percentage (1% to 8%) of candiduric patients develop candidemia and that ICU patients with candiduria are at the highest risk of becoming candidemic [7,8,28,29].

The differentiation between upper and lower urinary tract infections has been inherently difficult to make. A small imaging study, using white blood cells labeled with indium-111, concluded that 50% of the studied patients (n = 8) with candiduria showed renal uptake in ^111^In-labeled leukocyte scintigraphy, with uptake persisting after antifungal treatment [30]. This study excluded patients with concomitant bacteriuria, patients on antifungal treatment, and patients in the ICU setting. This finding should be confirmed in a larger study, as it raises the concern that subclinical pyelonephritis may be more frequent in patients with candiduria than thought. Data from experiments with rabbits suggest that the detection of renal *Candida* casts may be a useful diagnostic marker in distinguishing upper versus lower urinary tract candidiasis [18], but the frequency of this finding is unknown [7,31,32]. One study suggests that the D-arabinitol/creatinine ratio could be used to differentiate between *Candida* pyelonephritis and colonization [33]. However, in that study, the clinical distinction between pyelonephritis and colonization was poorly documented. Prostatitis and epididymitis can also lead to candiduria [7,34]. They are more common in older or immunocompromised men and should be evident by careful clinical examination. In some cases, these patients develop an abscess in the tissue. Candiduria is rarely associated with pneumaturia, which is the result of emphysematous tissue invasion or perinephric abscess formation [7,35]. These complicated urinary tract infections are observed predominantly in diabetic patients [7,36,37,38,39] and can also occur in the setting of prostatitis and epididymitis. In summary, most patients with candiduria have few or no symptoms, which complicates treatment decisions [7].

## 5. Immune Responses to Candiduria

Although candiduria and vulvovaginal candidiasis affect neighboring mucosal surfaces, the anatomy of the mucosal surfaces and their local microenvironments are different. The vaginal microenvironment is an estrogen-controlled environment, while the bladder milieu has a high urea content. As opposed to the vagina, the urinary tract is sterile. Most candiduria manifests as cystitis or pyelonephritis as a result of an ascending infection [7].

In an ascending UTI, the first line of defense against the pathogen is the one provided through the mucosa of the urinary tract and antibodies which can inhibit microbial adherence to mucosal surfaces [7,40,41]. However, the response of the urinary tract mucosa to *Candida* has not been studied. The immunization, eliciting with such antibodies in the mucosal surface, has been shown to protect against bacterial UTIs in monkeys [42]. In women, a vaginally administered vaccine has proven to be protective against reinfections in phase II trials [43]. All of these studies have been done in bacterial UTI models, but Epa proteins of *Candida glabrata* have also been demonstrated to mediate adherence and could potentially be blocked by antibodies [7,44].

The mechanisms involved in the immunological defense related to Candiduria are not completely elucidated. Instead, the immunological response in candidemia are better studied.

Defensins are elements that are involved in the host response in UTI [7,45], and their protective role against oral microbes (including *Candida*) has only been studied in oral epithelia [7,46]. Other important proteins, such as the Tamm-Hosfall protein (THP), with immunomodulatory capacity in bacterial urinary tract infections, acts in preventing fungal adhesion to the bladder epithelium, in a healthy host, resulting in the flow of urine washing away *Candida* before it establishes bladder infection. Additionally, THP is a potential target of interest in preventing catheter-associated UTI in hospitalized individuals [7,47]. The identification and blood quantification of antibody-secreting cells, including their lymphocyte receptors, in patients with pyelonephritis, has been researched as an indicator of local immune response [7,48,49]. Although the serological response has been investigated, correlations with invasive disease have not been established in candiduria [7,50]. We must consider that *Candida* is a commensal in the vagina, but not in the urinary tract, and the mechanisms of local mucous immunity may not be the same [7]. In fact, there is a knowledge gap about how an immune response contributes to pathologies in urinary tract infections.

## 6. Future Perspectives of Urinalysis on Fungal Urinary Tract Infections

Automated systems to perform urinalysis (especially equipment to perform urine particles identification and quantification) are evolving rapidly. They are based either on flow cytometry technology or the digital image method and have overcome the limitations of the manual microscopy methods (labor intensive, time-consuming procedures with a high interobserver variation) [51].

Enko et al. [51], evaluating both flow cytometry technology and the digital image method, compared the automated systems to phase contrast microscopy (the gold-standard method) and found sensitivity and specificity of 89.5% and 97.7%, and 31.6% and 96%, respectively, to the flow cytometry and digital image to yeast identification [51]. Despite the fact the digital image method presented results suggesting it needs improvement on the proper identification of yeasts, the flow cytometry technology showed good performance on the identification of this urinary particle.

Cho et al. [52], evaluating five different urinalysis automated systems (including both systems evaluated in the Enko work), found 44.4% sensitivity and 97.1% specificity to identify yeasts by flow cytometry, and 58.3% sensitivity and 95.7% specificity to identify yeasts by the digital image [52]. The equipment performance was not the same as that observed by Enko et al. (at least to the flow cytometry technology). However, in both studies, the specificity results showed a good performance.

Based on these results and on the good-quality performance that basically all equipment presented to erythrocyte and leukocyte identification/count, it is reasonable to hypothesize that, in a few years, yeast identification in automated systems will present high-quality performance. Regarding properly collected samples, the identification of yeasts and other particles related to fungal UTIs will be easily available using urinalysis automated systems. Also, the use of artificial intelligence, creating algorithms based on clinical and literature information, can contribute to an improved performance of the new technologies that are in use and will be available within laboratories.

## 7. Conclusions

The microscopic examination of the fresh and unstained urine sediment can contribute to the diagnosis of fungal UTI. However, positive findings should not be overinterpreted. Despite the knowledge about the immune response associated with candidemia, there is a knowledge gap on how immune responses contribute to the pathologies in fungal UTI. The future of the diagnosis of fungal UTI probably will be improved by the use of automated systems that will be able to properly identify if the urinary finding of yeasts/pseudohyphae is linked to a fungal UTI or if it is a sample contaminant.

## References

[1] Gharanfoli A., Mahmoudi E., Torabizadeh R., Katiraee F., Faraji S. (2019). Isolation, characterization, and molecular identification of *Candida* species from urinary tract infections. Curr. Med. Mycol..

[2] Kauffman C.A. (2014). Diagnosis and management of fungal urinary tract infection. Infect. Dis. Clin. N. Am..

[3] Reilly R., Perazella M.A. (2013). Nephrology in 30 Days.

[4] Fogazzi G.B. (2010). The Urinary Sediment—An Integrated View.

[5] Fisher F.J., Kavanagh K., Sobel J.D., Kauffman C.A., Newman C.A. (2011). *Candida* Urinary Tract Infection: Pathogenesis. Clin. Infect. Dis..

[6] Odabasi Z., Mert A. (2019). *Candida* urinary tract infections in adults. World J. Urol..

[7] Achkar J.M., Fries B.C. (2010). *Candida* infections of the genitourinary tract. Clin. Microbiol. Rev..

[8] Kauffman C.A., Vazquez J.A., Sobel J.D., Gallis H.A., McKinsey D.S., Karchmer A.W., Sugar A.M., Sharkey P.K., Wise G.J., Mangi R. (2000). Prospective multicenter surveillance study of funguria in hospitalized patients. The National Institute for Allergy and Infectious Diseases (NIAID) Mycoses Study Group. Clin. Infect. Dis..

[9] Fisher J.F., Sobel J.D., Kauffman C.A., Newman C.A. (2011). *Candida* urinary tract infections: Treatment. Clin. Infect. Dis..

[10] Dias V. (2020). *Candida* species in the urinary tract: Is it a fungal infection or not?. Future Microbiol..

[11] Gajdács M., Dóczi I., Ábrók M., Lázár A., Burián K. (2019). Epidemiology of candiduria and *Candida* urinary tract infections in inpatients and outpatients: Results from a 10-year retrospective survey. Cent. Eur. J. Urol..

[12] Colodner R.Y., Nuri Y., Chazan B., Raz R. (2018). Community-acquired and hospital-acquired candiduria: Comparison of prevalence and clinical characteristics. Eur. J. Clin. Microbiol. Infect. Dis..

[13] Perry J.D., Butterworth A., Nicholson M.R.A., Orr K.E. (2003). Evaluation of a new chromogenic medium, Uriselect 4, for the isolation and identification of urinary tract pathogens. J. Clin. Pathol..

[14] Jain N., Kohli R., Cook E., Gialanella P., Chang T., Fries B.C. (2007). Biofilm formation by and antifungal susceptibility of *Candida* isolates from urine. Appl. Environ. Microbiol..

[15] Okulicz J.F., Rivard R.G., Conger N.G., Nguyen M.X., Hospenthal D.R. (2008). Primary isolation of *Candida* species from urine specimens using chromogenic medium. Mycoses.

[16] Chabasse D. (2001). Yeast count in urine. Review of the literature and preliminary results of a multicenter prospective study carried out in 15 hospital centers. Ann. Françaises d Anesthésie et de Réanimation.

[17] Fazeli A., Kordbacheh P., Nazari A., Daie Ghazvini R., Mirhendi H., Safara M., Bakhshi H., Yaghoubi R. (2019). Candiduria in Hospitalized Patients and Identification of Isolated *Candida* Species by Morphological and Molecular Methods in Ilam, Iran. Iran. J. Public Health.

[18] Navarro E.E., Almario J.S., King C., Bacher J., Pizzo P.A., Walsh T.J. (1994). Detection of *Candida* casts in experimental renal candidiasis: Implications for the diagnosis and pathogenesis of upper urinary tract infection. J. Med. Vet. Mycol..

[19] Poloni J.A., Rotta L.N., Voegeli C.F., Pasqualotto A.C. (2013). *Cryptococcus* within a urinary cast. Kidney Int..

[20] Tesser Poloni J.A., Perazella M.A., Neild G.H. (2013). Macrophages at work: Phagocytosis of urinary fungi. Clin. Kidney J..

[21] Itoh K., Asai H.M.S., Nozaki T. (2014). Atlas of Urinary Sediment.

[22] Poloni J.A., Meinerz G., Monteiro Ade A., Keitel E., Rotta L.N. (2016). *Klebsiella pneumoniae* ESBL forming spheroplasts in the fresh and unstained urine sediment. J. Bras. Nefrol..

[23] Nikler A., Radišić Biljak V., Čičak H., Marić N., Bejuk D., Poloni J.A.T., Simundic A.M. (2019). *Escherichia coli* spheroplasts in a Croatian patient misclassified by two urine sediment analysers as erythrocytes: Case report. Biochem. Med. (Zagreb).

[24] Richards M.J., Edwards J.R., Culver D.H., Gaynes R.P. (2000). Nosocomial infections in combined medical-surgical intensive care units in the United States. Infect. Control Hosp. Epidemiol..

[25] Richards M.J., Edwards J.R., Culver D.H., Gaynes R.P. (1999). Nosocomial infections in pediatric intensive care units in the United States. National Nosocomial Infections Surveillance System. Pediatrics.

[26] Alvarez-Lerma F., Nolla-Salas J., Leon C., Palomar M., Jorda R., Carrasco N., Bobillo F. (2003). Candiduria in critically ill patients admitted to intensive care medical units. Intensive Care Med..

[27] Bougnoux M.E., Kac G., Aegerter P., D’Enfert C., Fagon J.-Y. (2008). Candidemia and candiduria in critically ill patients admitted to intensive care units in France: Incidence, molecular diversity, management and outcome. Intensive Care Med..

[28] Safdar N., Slattery W.R., Knasinski V., Gangnon R.E., Li Z., Pirsch J.D., Andes D. (2005). Predictors and outcomes of candiduria in renal transplant recipients. Clin. Infect. Dis..

[29] Bouza E., Juan R.S., Muñoz P., Voss A., Kluytmans J. (2001). A European perspective on nosocomial urinary tract infections II. Report on incidence, clinical characteristics and outcome (ESGNI-004 study). European Study Group on Nosocomial Infection. Clin. Microbiol. Infect..

[30] Gutierrez-Cuadra M., Horcajada J., Martinez I. (2007). 111-Indium labelled leukocyte renal scintigraphy in patients with candiduria: Preliminary results of a prospective study. Int. J. Antimicrob. Agents.

[31] Argyle C., Schumann G.B., Genack L., Gregory M. (1984). Identification of fungal casts in a patient with renal candidiasis. Hum. Pathol..

[32] Gregory M.C., GSchumann G.B., Schumann J.L., Argyle J.C. (1984). The clinical significance of candidal casts. Am. J. Kidney Dis..

[33] Tokunaga S., Ohkawa M., Takashima M., Hisazumi H. (1992). Clinical significance of measurement of serum D-arabinitol levels in candiduria patients. Urol. Int..

[34] Wise G.J., Shteynshlyuger A. (2006). How to diagnose and treat fungal infections in chronic prostatitis. Curr. Urol. Rep..

[35] Sultana S.R., McNeill S.A., Phillips G., Byrne D.J. (1998). Candidal urinary tract infection as a cause of pneumaturia. J. R. Coll. Surg. Edinb..

[36] Donders G.G. (2002). Lower genital tract infections in diabetic women. Curr. Infect. Dis. Rep..

[37] High K.P., Quagliarello V.J. (1992). Yeast perinephric abscess: Report of a case and review. Clin. Infect. Dis..

[38] Ronald A., Ludwig E. (2001). Urinary tract infections in adults with diabetes. Int. J. Antimicrob. Agents.

[39] Stapleton A. (2002). Urinary tract infections in patients with diabetes. Am. J. Med..

[40] Svanborg-Eden C., Svennerholm A.M. (1978). Secretory immunoglobulin A and G antibodies prevent adhesion of *Escherichia coli* to human urinary tract epithelial cells. Infect. Immun..

[41] Svanborg Eden C., Andersson B., Hagberg L., Hanson L.A., Leffler H., Magnusson G., Noori G., Dahmen J., Soderstrom T. (1983). Receptor analogues and anti-pili antibodies as inhibitors of bacterial attachment in vivo and in vitro. Ann. N. Y. Acad. Sci..

[42] Uehling D.T., James L.J., Hopkins W.J., Balish E. (1991). Immunization against urinary tract infection with a multi-valent vaginal vaccine. J. Urol..

[43] Uehling D.T., Hopkins W.J., Balish E., Xing Y., Heisey D.M. (1997). Vaginal mucosal immunization for recurrent urinary tract infection: Phase II clinical trial. J. Urol..

[44] Domergue R., Castaño I., Peñas A.D.L., Zupancic M., Lockatell V., Hebel J.R., Johnson D., Cormack B.P. (2005). Nicotinic acid limitation regulates silencing of *Candida* adhesins during UTI. Science.

[45] Zasloff M. (2007). Antimicrobial peptides, innate immunity, and the normally sterile urinary tract. J. Am. Soc. Nephrol..

[46] Abiko Y., Saitoh M., Nishimura M., Yamazaki M., Sawamura D., Kaku T. (2007). Role of beta-defensins in oral epithelial health and disease. Med. Mol. Morphol..

[47] Coady A., Ramos A.R., Olson J., Nizet V., Patrasa K.A. (2018). Tamm-Horsfall Protein Protects the Urinary Tract against *Candida albicans*. Infect. Immun..

[48] Kantele A., Palkola N., Arvilommi H., Honkinen O., Jahnukainen T., Mertsola J., Kantele J.M. (2008). Local immune response to upper urinary tract infections in children. Clin. Vaccine Immunol..

[49] Kantele A.M., Palkola N.V., Arvilommi H.S., Kantele J.M. (2008). Distinctive homing profile of pathogen-specific activated lymphocytes in human urinary tract infection. Clin. Immunol..

[50] Torres-Rodriguez J.M., Madrenys-Brunet N., Nolla-Salas J., Carceller A., Tur C. (1997). Candiduria in non-neutropenic critically-ill surgical patients. Detection of IgA, IgG and IgM antibodies to *Candida albicans* by germ tube immunofluorescence. Mycoses.

[51] Enko D., Stelzer I., Böckl M., Derler B., Schnedl W.J., Anderssohn P., Meinitzer A., Herrmann M. (2020). Comparison of the diagnostic performance of two automated urine sediment analyzers with manual phase-contrast microscopy. Clin. Chem. Lab. Med..

[52] Cho J., Oh K.J., Jeon B.C., Lee S.G., Kim J.H. (2019). Comparison of five automated urine sediment analyzers with manual microscopy for accurate identification of urine sediment. Clin. Chem. Lab. Med..

